# The effect of intravitreal brolucizumab on choroidal thickness in patients with neovascular age-related macular degeneration

**DOI:** 10.1038/s41598-022-23392-6

**Published:** 2022-11-18

**Authors:** Ki Woong Bae, Dong Ik Kim, Daniel Duck-Jin Hwang

**Affiliations:** 1 Department of Ophthalmology, Hangil Eye Hospital, #35 Bupyeong-Daero, Bupyeong-Gu, Incheon, 21388 Korea; 2 Department of Ophthalmology, Catholic Kwandong University College of Medicine, Incheon, Korea

**Keywords:** Eye diseases, Macular degeneration

## Abstract

In this study we evaluated the effect of intravitreal brolucizumab injections on choroidal thickness in patients with neovascular age-related macular degeneration (nAMD) who previously showed an incomplete response to anti-vascular endothelial growth factor treatment. A total of thirty-four eyes from 34 patients were included in this study. The patients received an average of 2.4 ± 1.1 brolucizumab injections with the mean follow-up period of 4.9 ± 2.0 months. After their first brolucizumab treatment, the central foveal thickness (CFT) and subfoveal choroidal thickness (SFCT) were significantly decreased from 431.6 ± 190.0 μm and 193.9 ± 75.1 μm to 274.6 ± 109.4 μm (*P* < 0.001) and 169.4 ± 71.1 μm (*P* < 0.001), respectively. However, there were no improvements in visual acuity. Patients were divided into three subgroups according to the number of brolucizumab treatments: one, two, and three or more injections. In all three subgroups, the CFT and SFCT were significantly reduced compared to baseline at all time points of brolucizumab injections. In conclusion, choroidal thickness was significantly reduced after intravitreal brolucizumab injections as a switching treatment in patients with nAMD.

## Introduction

Age-related macular degeneration (AMD) is one of the leading cause of blindness worldwide^1^. Neovascular AMD (nAMD) is characterized by retinal vascular leakage and fluid accumulation associated with choroidal neovascularization (CNV)^2^. To date, various treatment modalities have been attempted to inhibit CNV-induced exudation. Currently, intravitreal injection of anti-vascular endothelial growth factor (VEGF) agents is the first-line treatment for nAMD.

Brolucizumab is the most recently developed anti-VEGF agent used for nAMD^3^. Brolucizumab is the smallest molecules among the available anti-VEGF drugs and can be administered at higher concentrations than other anti-VEGF agents such as ranibizumab or aflibercept^4^. According to two pivotal clinical trials, including HAWK and HARRIER, brolucizumab was not inferior to aflibercept in terms of visual outcomes and demonstrated more favorable anatomical effects in post hoc analyses^3,5,6^.

Intravitreal injections of anti-VEGF agents may affect choroidal thickness. It has been reported that aflibercept reduced choroidal thickness to a greater extent than ranibizumab^7–9^. Koizumi et al. showed that the decrease in choroidal thickness from aflibercept treatment was associated with better visual and anatomic outcomes at one year^10^. However, a thinner choroid may be related to macular atrophy in long-term follow-up, which could result in severe vision loss^11,12^. Therefore, it is important to monitor the change of choroidal thickness during anti-VEGF treatment.

Some case series studies found that intravitreal brolucizumab injection reduced choroidal thickness^12–15^. However, little is known about the change in choroidal thickness of patients with nAMD who have already been treated with other anti-VEGF agents and switched to brolucizumab. Therefore, we evaluated temporal changes of subfoveal choroidal thickness (SFCT) in patients with nAMD who were treated with brolucizumab as a switching therapy due to an incomplete response to previous treatment.

## Results

### Baseline characteristics

The baseline characteristics of the 34 patients with nAMD are summarized in Table 1. Among them, 27 were male (79.4%) and the mean age was 70.6 ± 6.9 years. Polypoidal choroidal vasculopathy (PCV) was the most frequent AMD subtype (26 eyes, 76.5%), followed by typical AMD (6 eyes, 17.6%) and retinal angiomatous proliferation (RAP) (2 eyes, 5.9%). The mean number of brolucizumab injections was 2.4 ± 1.1, ranged 1–4 times. All eyes were non-treatment-naïve, and the mean number of previous anti-VEGF (non-brolucizumab) injections was 17.8 ± 10.1 (range, 3–40). The mean follow-up period was 4.9 ± 2.0 months (range, 1.8–8.0).

**Table 1:** Demographic and baseline characteristics (n = 34).

Age (years)	70.6 ± 6.9 (range, 56–85)
Sex, male: female	27:7
Type of AMD (N, %) PCV: typical AMD: RAP	26 (76.5), 6 (17.6), 2 (5.9)
Previous number of intravitreal injections	17.8 ± 10.1 (range, 3–40)
Injection number of brolucizumab	2.4 ± 1.1 (range, 1–4)
Adverse events (N, %)	5 (14.7)
Mean follow up period (months)	4.9 ± 2.0 (range, 1.8–8.0)
Hypertension (N, %)	14 (41.2)
Diabetes mellitus (N, %)	7 (20.6)
Spherical equivalent (diopters)	+ 0.44 ± 1.35 (range,− 3.00 − + 2.75)
Corrected visual acuity (logMAR)	0.42 ± 0.27 (range, 0.05–0.82)
IOP (mmHg)	14.2 ± 2.5

Values are presented as N (percentage) or mean ± standard deviation.

*AMD* age-related macular degeneration; *PCV* polypoidal choroidal vasculopathy; *RAP* retinal angiomatous proliferation; *logMAR* logarithm of the minimal angle of resolution; *IOP* intraocular pressure.

### Visual outcomes and central foveal thickness (CFT) after intravitreal brolucizumab injection

At the baseline, the average best corrected visual acuity (BCVA), converted to the logarithm of the minimal angle of resolution (logMAR), was 0.42 ± 0.27 (range 0.05–0.82) and 0.42 ± 0.32 (range 0.05–1.30) (*P* = 0.921) one month after the first injection. There was no significant change in vision, even after additional brolucizumab treatment.

Among the 34 study eyes, the initial CFT was 431.6 ± 190.0 μm, which significantly decreased to 274.6 ± 109.4 μm (*P* < 0.001) after the first brolucizumab treatment. The contralateral 17 eyes that had no retinal pathology including epiretinal membrane, age-related macular degeneration, macular hole, or history of vitrectomy, had an average initial CFT of 286.2 ± 52.6 μm and SFCT of 246.5 ± 65.3 μm during the same period with no significant change at one month follow up.

**Figure 1:**
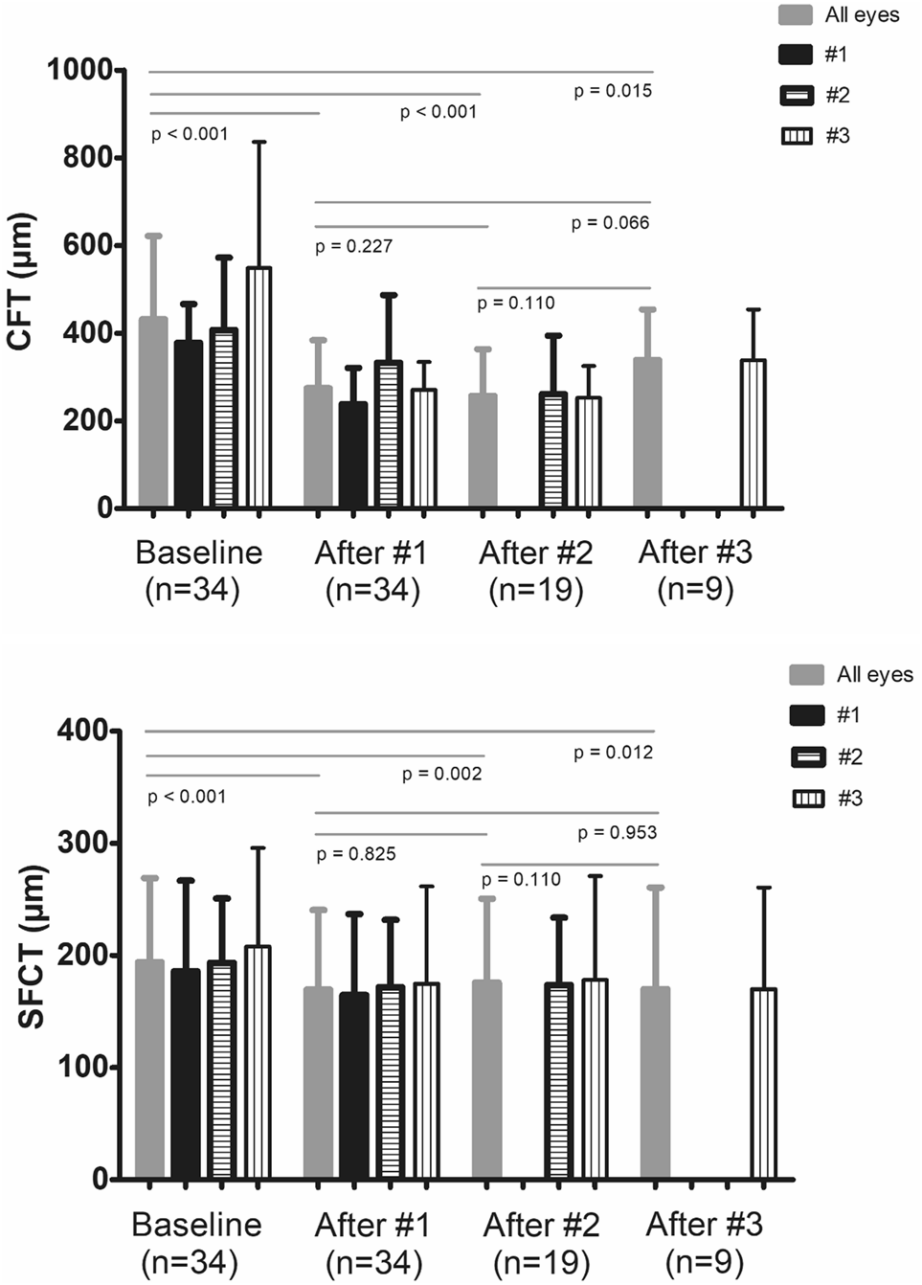
Change of central foveal thickness (CFT) and subfoveal choroidal thickness (SFCT) after intravitreal brolucizumab treatment stratified by number of injections. Data were presented with mean and standard deviation. Eyes were divided into three groups according to the number of brolucizumab injections. CFT and SFCT were significantly decreased after brolucizumab injection compared to the baseline in all three groups (*P* < 0.05). *P* value was obtained from paired T test or Wilcoxon signed rank test.

**Figure 2:**
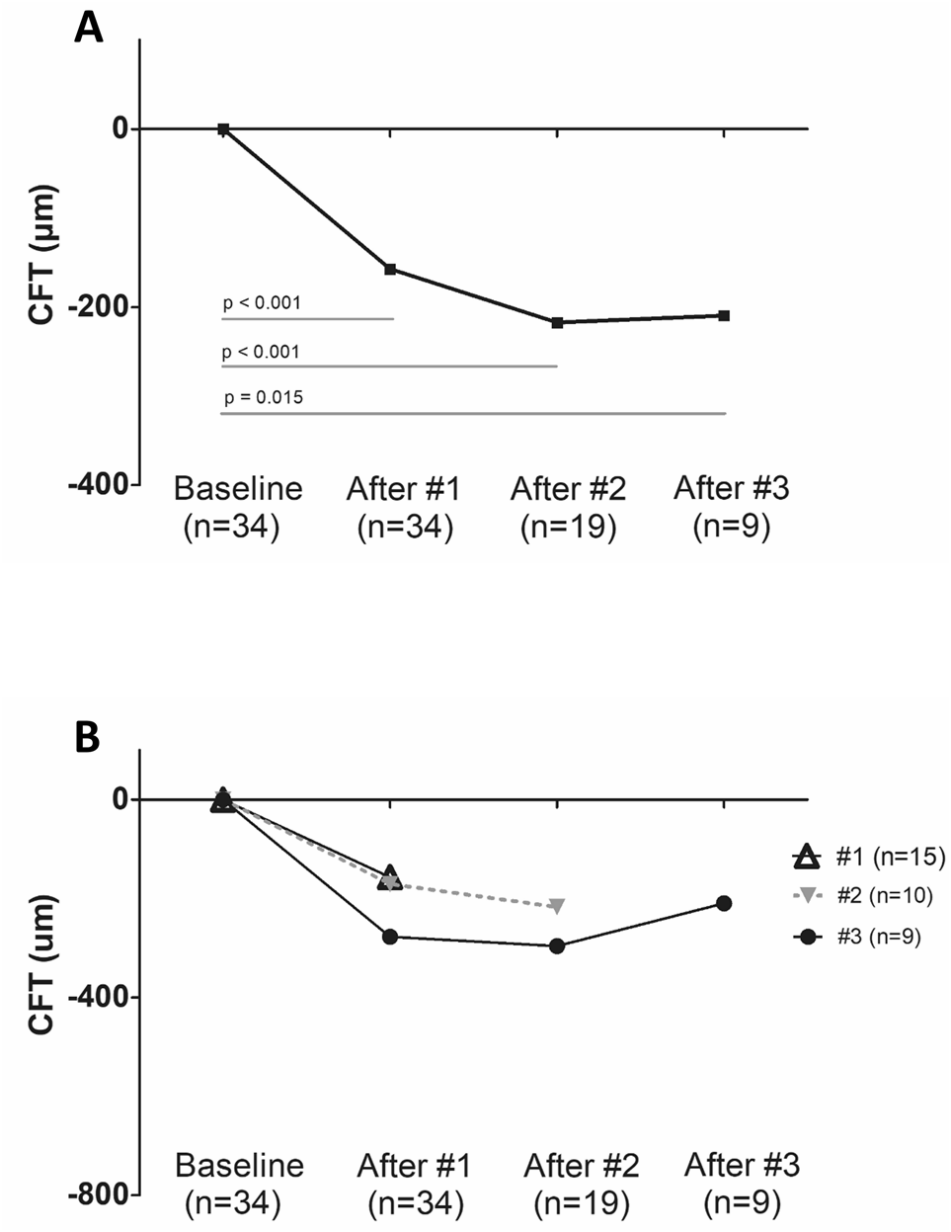
Temporal change in central subfoveal thickness (CFT) compared to baseline stratified by number of intravitreal brolucizumab injections. (**A**) Data presented as the average. The mean CFT of all patients was significantly decreased compared to baseline at all three-timepoints; after one, two, and three injections (*P* < 0.05). (**B**) Eyes were divided into three groups according to the number of brolucizumab injections. The CFT was significantly decreased after brolucizumab injection compared to the baseline in all three groups (*P* < 0.05). However, there was no significant change in the CFT between the time of #1 injection and #2 injections in patients with two injections of brolucizumab (n = 10, *P* = 0.169). Also, there was no significant change in the CFT in subjects with three injections of brolucizumab among three time points (n = 9); at the time of #1 injection and #2 injections (*P* = 0.953), #1 injection and #3 injections (*P* = 0.066), and #2 injections and #3 injections (*P* = 0.110), respectively. *P* value was obtained from paired T test or Wilcoxon signed rank test.

**Figure 3:**
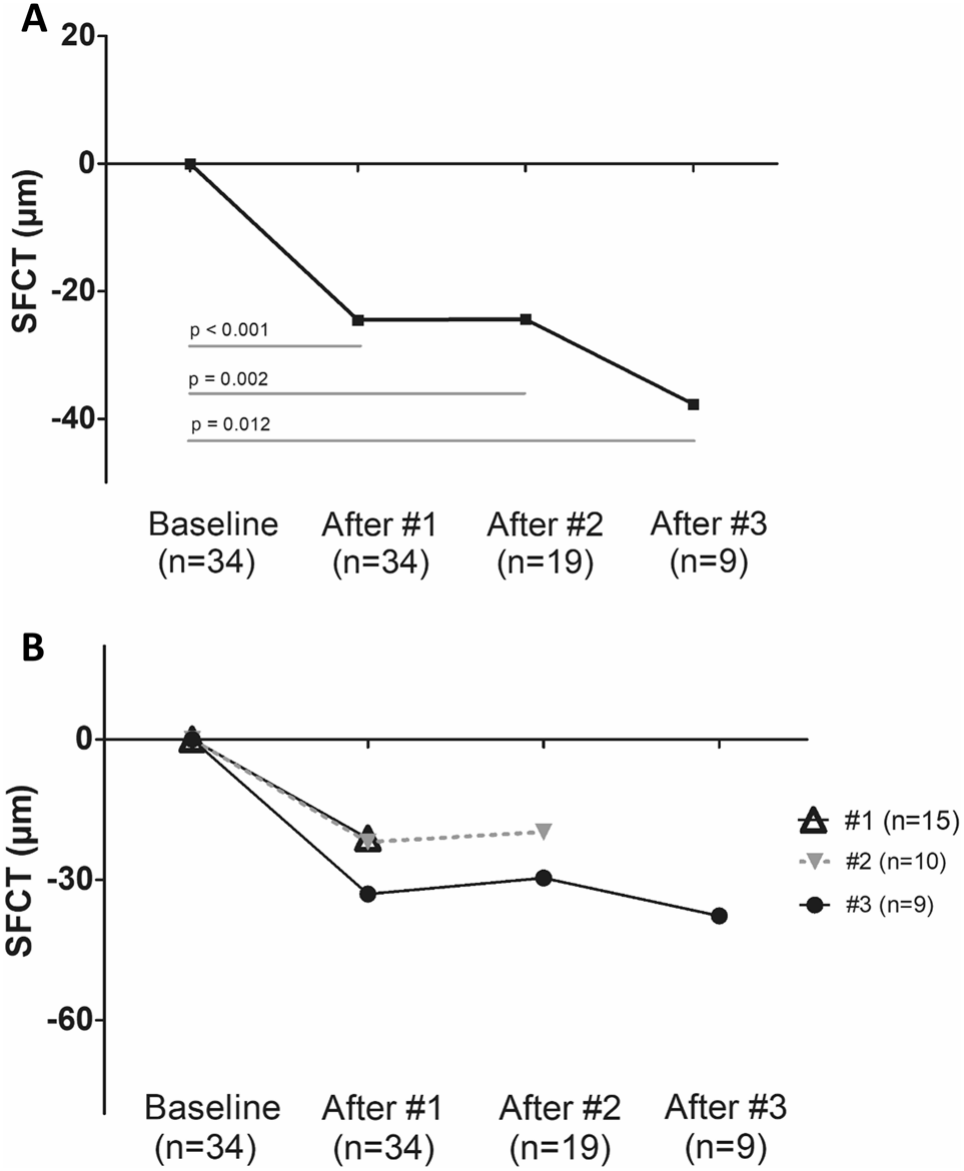
Temporal change in subfoveal choroidal thickness (SFCT) compared to baseline stratified by number of intravitreal brolucizumab injections. (**A**) Data were presented as the average. The mean SFCT of all patients significantly decreased compared to baseline at all three-timepoints; after one, two, and three injections (*P* < 0.05). (**B**) Eyes were divided into three groups according to the number of brolucizumab injections. The SFCT was significantly decreased after brolucizumab injection compared to the baseline in all three groups (*P* < 0.05). However, there was no significant change in the SFCT between the time of #1 injection and #2 injections in patients with two injections of brolucizumab (n = 10, *P* = 0.799). Also, there was no significant change in the SFCT in patients with three injections of brolucizumab among three time points (n = 9); at the time of #1 injection and #2 injections (*P* = 0.441), #1 injection and #3 injections (*P* = 0.953), and #2 injections and #3 injections (*P* = 0.110), respectively. *P* value was obtained from paired T test or Wilcoxon signed rank test.

The temporal changes in retinal thickness are presented in Figs. 1, 2, and 3. The CFT was significantly decreased after additional brolucizumab injections compared to baseline CFT; after the second and third brolucizumab injections, the CFTs were 256.8 ± 106.4 μm (n = 19, *P* < 0.001) and 338.8 ± 115.4 μm (n = 9, *P* = 0.015), respectively.

### Temporal changes in subfoveal choroidal thickness after intravitreal brolucizumab injection

**Figure 4:**
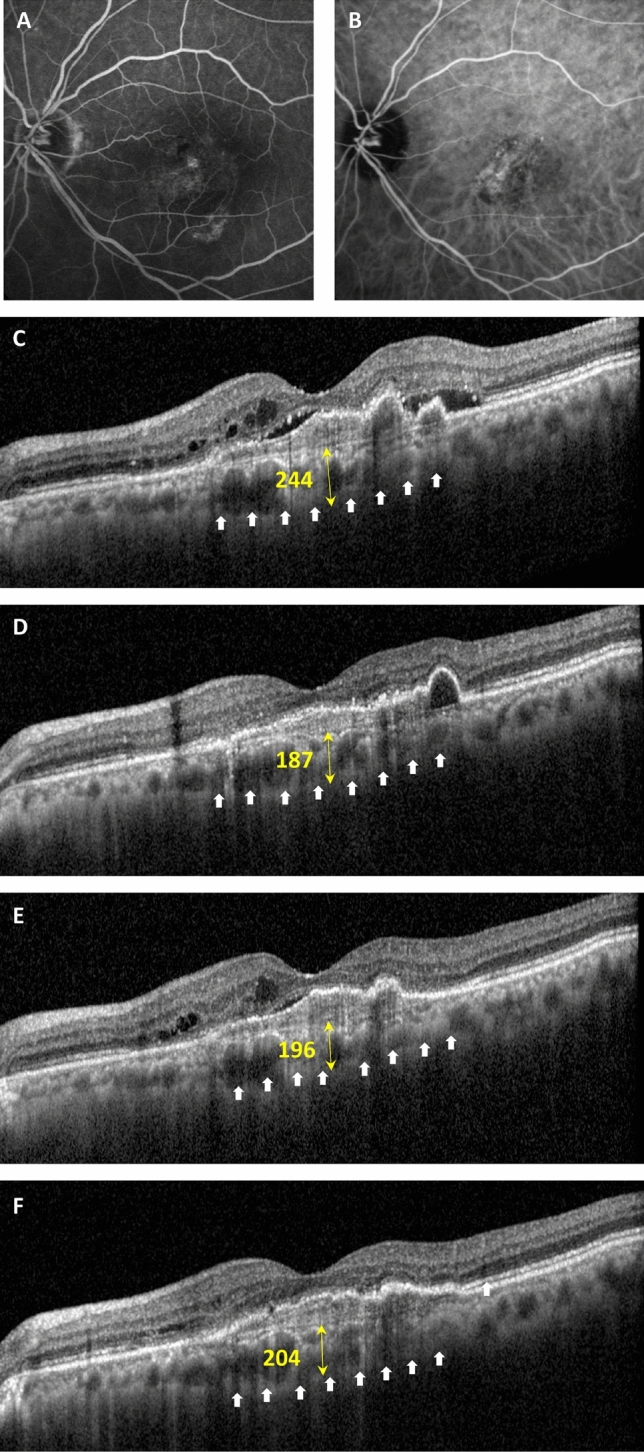
A representative case of a 69 year old male with polypoidal choroidal vasculopathy. The patient had previously received more than 10 treatments with ranibizumab and aflibercept, however macular exudation was persistent and treatment agent was switched to brolucizumab. (**A**) Fluorescein angiography and (**B**) indocyanine green angiography showing a subfoveal polyp with a branching vascular network. (**C**) Optical coherence tomography (OCT) at baseline showing fibrovascular retinal pigment epithelium detachment accompanied with intra and subretinal fluid. The white arrows indicate the inner scleral border and yellow arrows demonstrate the subfoveal choroidal thickness (SFCT). Central foveal thickness (CFT) was 333 μm and SFCT was 244 μm. (**D**) One month after the first brolucizumab treatment, CFT and SFCT were decreased to 253 and 187 μm, respectively. Complete fluid resolution was presented in the OCT image. Best corrected visual acuity (BCVA) also improved from 0.5 to 0.2 logarithm of the minimal angle of resolution (logMAR). No adverse reactions related with brolucizumab injection were reported. (**E**) Three months after first injection, intra and subretinal fluid was found in OCT scans and an additional injection was performed. CFT and SFCT were 317 and 196 μm and both were still reduced compared to baseline. (**F**) At last visit, six months after baseline, mild intraretinal fluid was remained and a third brolucizumab injection was administrated. BCVA was 0.15 logMAR. CFT and SFCT were 300 and 204 μm, respectively.

For 34 eyes, the mean SFCT before brolucizumab treatment was 193.9 ± 75.1 μm which decreased 12.7% from the baseline to 169.4 ± 71.1 μm (*P* < 0.001) after the first injection. The SFCT was significantly decreased after additional brolucizumab injections compared to baseline SFCT; after the second and third brolucizumab treatments to 175.8 ± 74.9 μm (n = 19, *P* = 0.002) and 170.1 ± 90.5 μm (n = 9, *P* = 0.012), respectively. Figure 4 shows an example of brolucizumab treatment.

### Adverse events

Among the 34 eyes, adverse events were reported in five eyes (14.7%) of five patients (6.0% from the total 83 injections) during the observation period. Of the five eyes with intraocular inflammation (IOI), three eyes showed only anterior uveitis and two eyes showed additional signs of vitreous opacity and occlusive vasculitis. Treatment for IOI was adjusted according to the severity of the adverse events. In cases with only mild IOI without signs of posterior uveitis or vasculitis, topical corticosteroids were initiated and tapered. Two patients with retinal vasculitis were treated with local and systemic corticosteroids. All five patients with IOI recovered, and none of them had persistent visual disturbances.

## Discussion

We evaluated the effect of brolucizumab on choroidal thickness in patients with nAMD who previously had an incomplete response to other anti-VEGF agents. The CFT and SFCT significantly decreased after the first brolucizumab injection, while BCVA did not show improvement compared with baseline. Patients were divided into three subgroups according to the number of brolucizumab treatments: one, two, and three or more injections. In all three subgroups, the CFT and SFCT were significantly reduced compared to baseline at all time points of brolucizumab injections.

Yun et al. reported a study in which three monthly injections of aflibercept or ranibizumab were administered to patients with nAMD^9^. They showed a significant reduction in choroidal thickness in both groups and the change was greater in the aflibercept group. Recently, some reports have shown changes of choroidal thickness in patients with nAMD after brolucizumab treatment. Fukuda et al. performed a study comparing the effects of three monthly aflibercept or brolucizumab injections in 52 treatment-naïve PCV patients^13^. In both groups, the choroidal thickness was significantly reduced by 35.6 μm and 38.9 μm, respectively. However, this difference was not significant between two groups. Matsumoto et al. evaluated the effect of brolucizumab injections in 42 eyes with treatment-naïve type 1 CNV^14^. The patients received three monthly injections of brolucizumab. The choroidal thickness decreased by 10.6% after the first injection and by 15.5% after three injections. Ota et al. investigated the effect of brolucizumab in patients with nAMD who showed resistance to aflibercept treatment^15^. After the first brolucizumab injection, choroidal thickness decreased by 12.9 μm (7.1%). Tamashiro et al. reported the results of three monthly injections of brolucizumab in patients with nAMD^12^. The subjects were divided into two subgroups: treatment-naïve and switched. Choroidal thickness decreased significantly in both groups, and the degree of change was greater in the treatment-naïve group (36.1 μm, 15.6% vs. 12.1 μm, 5.3%).

In this study, we included patients with nAMD who were treated with brolucizumab as a switching treatment. Unlike other studies where monthly injections were performed, the treatment interval was two or three months in this study population. For a total of 34 eyes, brolucizumab injection was administered at least once, 19 eyes at least twice, and 9 eyes at 3 or 4 times. Since the injection interval was longer than that in previous studies, we could evaluate the effect of brolucizumab on choroidal thickness for a relatively longer period, with an average of 4.9 months and up to 8 months. The reduction in choroidal thickness in this study was 12.7%, which was generally similar to that reported in previous studies. This is lower than the results of studies from treatment-naïve patients, which may be because we included non-naïve patients who had been treated with other anti-VEGF agents.

Brolucizumab is the smallest molecules among the available anti-VEGF drugs and can be administered at higher concentrations^4^. Therefore, it could achieve a more favorable anatomical outcome than other anti-VEGF agents^5,6^. Previous studies on the effect of brolucizumab treatment in patients with nAMD showed a reduction in choroidal thickness. This study also confirmed that brolucizumab injections reduced choroidal thickness even in patients who were refractory to other anti-VEGF treatments. However, it is not clear whether this choroidal thickness reduction in nAMD is positive or negative for prognosis. Koizumi et al. reported that a decrease in choroidal thickness was related to improved visual outcomes after aflibercept injections^10^. In contrast, Sadda et al. showed that a thinner choroid was a risk factor for the development of macular atrophy^11^. Future studies are needed to determine the long-term effects of the changes in choroidal thickness after brolucizumab treatment.

This study has some limitations. The study design is retrospective and included a small number of patients. In addition, the AMD types were heterogeneous and all the subjects were Korean; therefore, the results may not be generalizable to other racial or ethnic groups. However, the observation period was up to 8 months, which is meaningful as it is the longest follow-up among studies that investigate the changes in choroidal thickness after brolucizumab treatment. In addition, we consecutively recruited the subjects who were treated with brolucizumab as a switching therapy. The enrolled patients had various AMD types and different treatment periods with other anti-VEGF agents. Therefore, this study showed real-world data from actual nAMD practice. In summary, choroidal thickness was significantly reduced after intravitreal brolucizumab injections as a switching treatment in patients with nAMD.

## Methods

This retrospective study was approved by the Institutional Review Board of Hangil Eye Hospital and adhered to the tenets of the Declaration of Helsinki. The requirement to obtain informed consent from study participants was waived by the institutional review board given the retrospective nature of the study.

### Patients

This retrospective, observational, consecutive case series study enrolled patients between April 2021 and December 2021. A total of thirty-four eyes from 34 patients with nAMD were included. Patients with geographic atrophy or disciform scars in the macula were excluded. Eyes with other ocular diseases, such as glaucoma, uveitis, and vitreoretinal disease, were also excluded. Previous ocular trauma or surgery, except for cataract extraction, were also exclusion criteria in this study. All patients had been previously treated with other anti-VEGF agents, such as bevacizumab, ranibizumab, and/or aflibercept. However, despite those previous frequent intravitreal injections, fluid accumulation persisted on spectral domain optical coherence tomography (SD-OCT), and another treatment option was needed. Therefore, anti-VEGF drug was switched to brolucizumab. The baseline visit was regarded as the day of the initial intravitreal brolucizumab injection. Medical records and SD-OCT results were reviewed at baseline, at the time of each brolucizumab injection, one month after treatment, and at the final visit.

PCV was diagnosed based on the presence of polypoidal lesions, with or without branching vascular networks. Cases that exhibited retinal-retinal or retinal-choroidal anastomoses were classified as type 3 neovascularization (RAP)^16,17^. The remaining patients who were not diagnosed with either PCV or RAP were classified as having typical nAMD with type 1 or type 2 CNV^18^.

### Protocols for optical coherence tomography (OCT) scans

SD-OCT examination with macular thickness mapping was performed using a Spectralis OCT (Heidelberg Engineering, Heidelberg, Germany). A central volume scan with a 25-scan pattern and macular thickness map protocol was performed. CFT was obtained from the central 1 mm subfield in the macular thickness map presented by the OCT machine. SFCT, defined as the perpendicular distance from Bruch’s membrane to the sclera-choroidal junction at the subfovea, was measured manually using a caliper provided by the OCT machine. The average thickness of the horizontal and vertical scans was defined as the SFCT^19^.

### Intravitreal injection methods

All injections were performed on the day of the OCT examination. Under topical anesthesia with proparacaine (0.5%) eye drops, the bulbar conjunctiva and fornices were rinsed with 5% povidone-iodine, followed by the application of a sterile drape and lid speculum. After a drop of 5% povidone-iodine was applied, 6 mg (0.05 mL) of brolucizumab was injected into the pars plana. The needle was removed carefully, and the injection site was compressed using a sterile cotton applicator to prevent reflux. Antibiotic eye drops were applied four times per day for one week after injection.

### Data analysis

Statistical analyses were performed using a commercially available software package (IBM SPSS Statistics 25.0; SPSS Inc.,Chicago, IL, USA). Data were analyzed by dividing them into continuous and descriptive variables. Quantitative variables were presented as mean ± standard deviation, and qualitative variables were reported as absolute frequencies and percentages. Temporal changes in BCVA and retinal thickness were also evaluated. Statistical significance was defined as *P* < 0.05.

## Data Availability

The data are not available for public access because of patient privacy concerns, but are available from the corresponding author upon reasonable request.
